# Research Progress and Prospects of Autophagy in the Mechanism of Multidrug Resistance in Tumors

**DOI:** 10.1155/2022/7032614

**Published:** 2022-01-30

**Authors:** Wenqing Long, Lijun Zhang, Yuxing Wang, Haijuan Xie, Lin Wang, Hongnu Yu

**Affiliations:** ^1^Department of Thoracic Surgery, Affiliated Xinhua Hospital of Dalian University, Dalian 116021, China; ^2^Department of Oncology, Affiliated Xinhua Hospital of Dalian University, Dalian 116021, China

## Abstract

Although the treatment of cancer has made great strides in clinical practice, its high morbidity and fatality rates remain a major threat to human health. Multidrug resistance (MDR) often appears in the process of tumor treatment, leading to tumor refractory and aggravating the risk of tumor recurrence. Therefore, antitumor MDR plays a key role in tumor chemotherapy. Autophagy is an important process for the turnover of intracellular materials, which is commonly seen in the treatment of sensitive and multidrug-resistant tumors, and it can play different roles in various types of MDR tumor cells and tissues. Autophagy plays a dual regulatory role in MDR tumors. On the one hand, autophagy can promote the formation of MDR in tumor cells, weaken the killing effect of chemotherapy drugs on tumor cells, and play a protective role in tumor survival. On the other hand, autophagy production in the cellular environment can kill MDR tumor cells, reverse tumor resistance and enhance the efficiency of chemotherapy drugs. Therefore, the regulation of autophagy to overcome MDR has become increasingly significant in tumor chemotherapy. In this article, we discussed and summarized the research progress of autophagy in MDR tumors, mainly involving the different characteristics of autophagy in MDR cancer cells.

## 1. Introduction

Cancer is a major challenge to human health and one of the most important causes of human death among many pathogenic factors. Nowadays, great advances have been made in the clinical treatment of cancer, including radiotherapy, chemotherapy, surgery, molecular targeted therapy, immunotherapy, and so on. There are still many challenges and difficulties in finding a cure for cancer, including cancer recurrence, metastasis, and MDR. These treatment challenges can lead to poor prognosis and high cancer mortality. MDR has become an important factor in the failure of cancer chemotherapy and can lead to recurrence and metastasis. Tumor MDR involves a variety of physiological processes in the cellular environment, including increased drug efflux, decreased uptake of drugs by the body, cell resistance to apoptosis, changed cell cycle points, changed drug effect targets, and eliminated drug toxicity, etc. [1]. In recent years, the molecular mechanisms of MDR have been extensively studied but not fully elucidated.

In normal cells, autophagy can play a role in degrading damaged organelles and misfolded proteins. While removing them, autophagy also participates in the physiological cell metabolism process and makes an important difference in growth and development, adaptation to starvation, cell death, and tumor inhibition [2, 3]. This prevents the accumulation of excess material in the cell and helps maintain homeostasis. More importantly, this process can release the large molecule materials required for the synthesis of new proteins [4]. In general, phagocytes are regarded as vesicles with scavenging abilities and are the structures that recruit autophagy-related proteins to induce autophagy. In the process of autophagy, the lipid membrane will prolong and form a complete and closed bilayer structure. Eventually, the autophagy lysosome is formed to degrade its encapsulated contents and recover nucleotides, amino acids, and other materials that can be recycled [5, 6]. Autophagy occurs frequently during tumorigenesis and chemotherapy. In addition, autophagy produced during chemotherapy can protect cancer cells from the effects of drug toxicity, leading to tumor refractory and drug resistance [7]. Interestingly, recent research mechanisms suggest that autophagy-related signaling pathways are involved in the occurrence and development of MDR [8]. In recent years, research has explored the methods of using autophagy to reverse MDR tumor cells in the related process of tumor therapy, but the relationship between autophagy and MDR has not been fully studied. Therefore, an in-depth study of the relationship between autophagy and the occurrence and development of MDR tumors and their regulatory mechanisms could provide a new idea for the clinical treatment of tumors. In this paper, the role and treatment of autophagy in MDR tumors are reviewed.

## 2. Autophagy

### 2.1. Definition of Autophagy and Its Physiological Effects

Autophagy is an ancient cellular decomposition process used to remove excess or dysfunctional organelles and large subcellular structures, thus playing an important housekeeping role in cells. Autophagy is extremely sensitive to nutrient supply and is upregulated at the transcriptional and posttranslational levels in response to nutrient deficiency. It also helps to promote the circulation of cell components and nutrients to maintain the growth and survival of cells [9]. Autophagy plays a beneficial and essential role in the physiological processes of the body, which prevents the formation of toxic and side-effecting protein aggregates and the accumulation of damaged organelles. The accumulation of these substances can lead to cell death, tissue damage, and even the occurrence of chronic inflammation in tumor tissue. Furthermore, autophagy can provide the necessary energy and substrate for intracellular material transport and the survival of an organism [10, 11]. Autophagy also plays a key role in various tissue processes, immune responses, and the regulation of inflammation [12].

### 2.2. Discovery of Autophagy-Related Genes

Genetic studies in yeast have laid the foundation for the preliminary discovery of autophagy-related genes and the study of molecular signaling pathways involved in the process of autophagy [13]. So far, more than 40 related genes involved in autophagy formation and regulation have been found. They are essential for coping with microenvironmental stresses such as heat stress, hypoxia, and the accumulation of reactive oxygen species (ROS). And they participate in the induction and initiation of autophagy, the extension of the autophagic membrane, and the mature degradation stages [14].

### 2.3. Molecular Mechanisms and Processes of Autophagy

**Figure 1:**
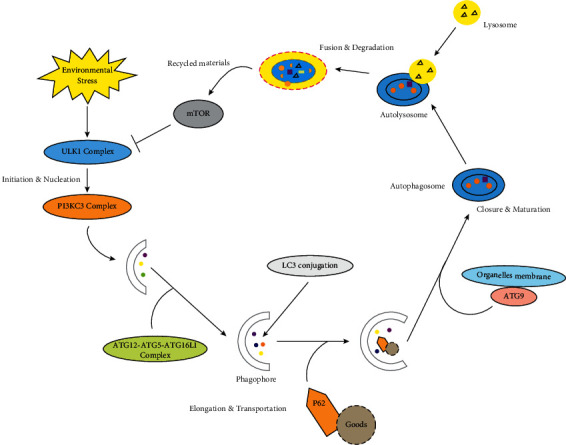
A schematic diagram of the autophagy process. This process consists of several phases: Initiation and Nucleation; Elongation and Transportation; Closure and Maturation; Fusion and Degradation. Under the stimulation of external environmental factors such as hypoxia, energy deficiency, and cell stress, etc. The initiation process starts with the activation of the ULK1 complex, and the PI3KC3 complex regulates nucleation. The ATG12-ATG5-ATG16L1 complex and LC3 conjugation participate in the elongation of the phagophore, and the ATG9 protein recruits organelles' membranes to form the autophagic vesicles. After maturation, the autophagosome fuses with the lysosome to form an autolysosome. The autolysosome contents are then degraded.

Because autophagy is a complicated multistep process, mastering the details of autophagy is crucial for researching effective drugs and therapies to regulate autophagy specifically and efficiently. In our understanding, the autophagy process can be separated into the steps in Figure 1.

#### 2.3.1. The ULK1 Complex Participates in the Initiation of Autophagy

The Unc-51-like Kinase 1 (ULK1) complex is composed of an autophagy-related protein 1 (ATG1) homolog from the ULK family (ULK1/2), ATG13, and focal adhesion kinase interacting protein 200 kDa (FIP200). In general, the complex is stable and inactive regardless of nutrient status [15]. However, the association of the mechanistic target of rapamycin complex1 (mTORC1) with the induction of the complex is influenced by nutrient status.

**Figure 2:**
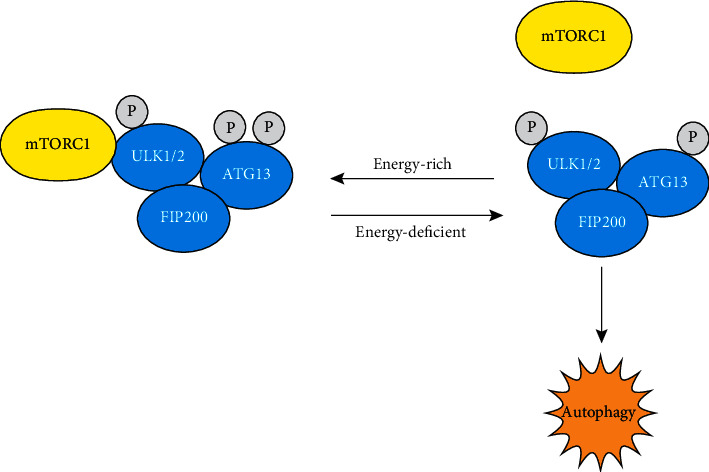
A schematic diagram of ULK1 complex induction. Under energy-deficient conditions, mTORC1 dissociates from the ULK1 complex, leaving ULK1/2 and ATG13 partially dephosphorylated, and then the complex can seduce autophagy. Under energy-rich conditions, mTORC1 combines with the complex and inactivates the ULK1/2 and ATG13 proteins across phosphorylation.

mTOR is a kind of serine/threonine (Ser/Thr) protein kinase [16], and it plays a significant regulatory role in autophagy. In mammals, mTOR kinase is present in two different complexes, including functional complexes: mTOR complex 1 (mTORC1) and complex 2 (mTORC2) [17]. In mTOR complexes, mTORC1 is more sensitive to rapamycin and can inhibit the initiation of autophagy by phosphorylating the ULK1 complex, while the ULK1 complex is an important forward regulatory factor in the process of autophagosome formation. Under energy-rich circumstances, mTORC1 combines with the ULK1 complex but dissociates under energy deficiency. When mTORC1 associates with the complex, it phosphorylates ULK1/2 and ATG13 to inactivate them. When the energy supply is insufficient, mTORC1 can be separated from the ULK1 complex, thus inducing the formation and extension of the autophagosome membrane and inhibiting cell proliferation (Figure 2).

#### 2.3.2. The PI3KC3 Complex Is Involved in Nucleation Regulation

After the ULK1 complex is activated, the class III phosphatidylinositol-3-kinase (PI3KC3) complex is recruited to the presumptive site of autophagosome formation. The PI3KC3 complex consists of PI3K vacuolar protein sorting 34 (Vps34), Vps15, ATG14L, and Beclin1 (BECN1), and it participates in the nucleation of the phagophore.

**Figure 3:**
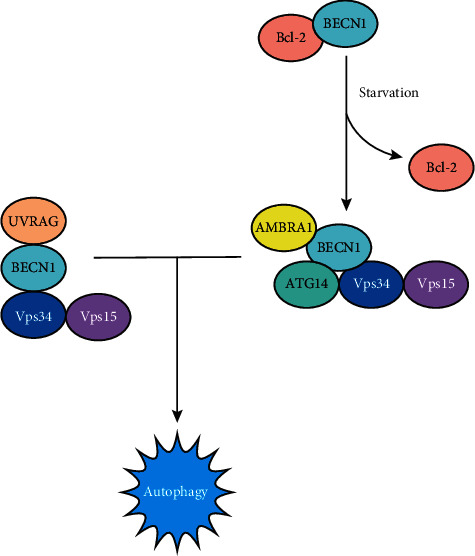
A schematic diagram of the PI3KC3 complex involved in the regulation of autophagy. The PI3KC3 complex is necessary for autophagy and is composed of BECN1, ATG14, Vps15, and Vps34. The complex can be positively regulated by AMBRA1 and negatively regulated by Bcl-2, which binds to BECN1 and blocks the combination with the complex. The UVRAG protein is a BECN1 positive mediator, and it can also mediate the activation of the PICKC3 complex to promote autophagy.

Some proteins, such as Vps34, autophagy and BECN1 regulator 1 (AMBRA1), and BECN1, have been identified as the ideal regulator proteins in the formation of the phagophore [18–21]. BECN1 is a member of the PI3KC3 complex. The complex is regulated primarily by proteins that mutually interact with BECN1, which is crucial for the complex and autophagy. BECN1 is mainly located in the trans-Golig network (TGN), endoplasmic reticulum, and mitochondria. Its activity is inhibited by the Bcl-2 protein family after binding to Bcl-2, which inhibits autophagy. Under the deficiency of energy, activated c-Jun N-terminal kinase 1 (JNK1) phosphorylates Bcl-2 and interferes with the interaction between Bcl-2 and BECN1. Dissociative BECN1 combines with Vps34 to form a PI3KC3 complex to induce membrane nucleation. In addition, many different mediators, such as ATG14L, AMBRA1 [22, 23], and UV radiation resistance-associated gene (UVRAG) [24], interact with BECN1 to differentially regulate membrane formation. The PI3KC3 complex can phosphorylate phosphatidylinositol to form phosphatidylinositol 3-phosphate (PI3P), and PI3P can mobilize other ATGs in the cytoplasm to bind to the membrane of the proautophagosome, which plays an important role in the early stage of autophagosome formation [25] (Figure 3).

#### 2.3.3. The ATG12-ATG5-ATG16L1 Complex and the LC3 Conjugation Cascade

**Figure 4:**
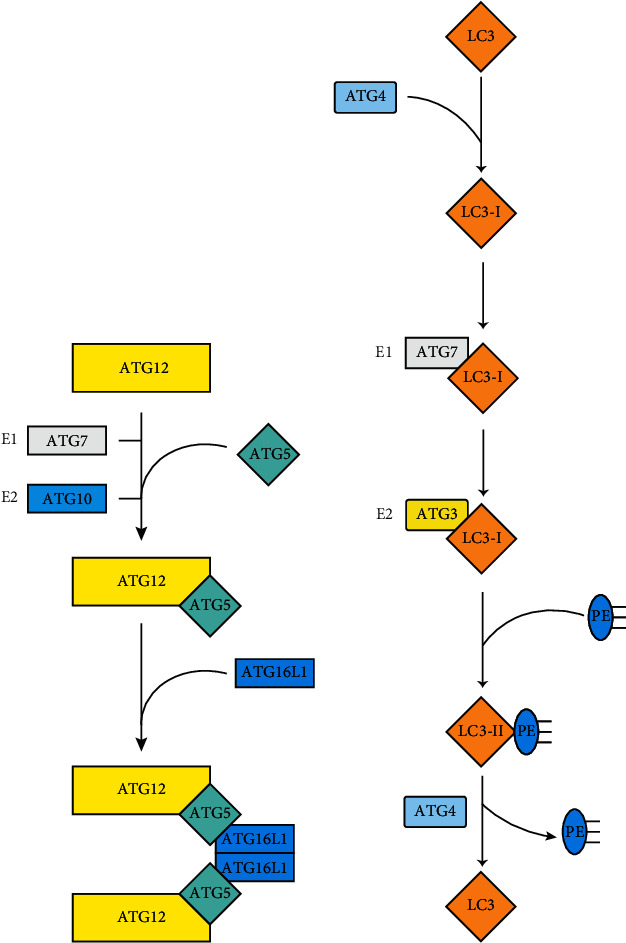
(a) The ATG12-ATG5-ATG16L complex. The conjunction of ATG12 and ATG5 begins with the activation of the E1-like enzyme ATG7 and the E2-like enzyme ATG10. Then ATG12-ATG5 combines with ATG16L1 through ATG5. Finally, ATG16L1 dimerizes and binds to the phagosome to promote membrane expansion. (b) The LC3 conjugation system. LC3 is cleaved by ATG4 to form LC3-I and exposes a C-terminal glycine, which can bind to PE. ATG7 is an E1-like enzyme that activates LC3-I and transports it to the E2-like enzyme ATG3. LC3-I is converted to LC3-II by interacting with PE. Eventually, LC3-II can be cleaved by ATG4 to release the PE and LC3.

The accumulation of proteins that contain the PI3P binding domain at the membrane nucleation site can lead to the integration of extra ATGs, which is necessary for the expansion and closure of the phagophore membrane. There are two ubiquitin-like (UBL) conjugation systems that can regulate the elongation of the membrane. In the first system, the E1-like enzymes ATG7 and E2-like enzymes ATG10 jointly catalyze the formation of an ATG12-ATG5 conjugated complex [26]. Finally, ATG16L1 interacts with the ATG12-ATG5 complex to form an ATG12-ATG5-ATG16L1 complex in a self-oligomerization manner, acting as an E3-like effect for the second UBL coupling system [27–29]. After the autophagy precursor membrane completely fuses to form closed autophagosomes, the ATG12-ATG5-ATG16L1 complex is released into the cytoplasm [30] (Figure 4(a)).

The second UBL system is composed of the conjugation between LC3 and PE. LC3 is involved in the formation of the autophagosome membrane and consists of two interchangeable forms: LC3-I and LC3-II. The intracellular synthesis of LC3 is processed into the cytoplasmic soluble form of LC3-I, during which ATG4 cleaves LC3 to form LC3-I and exposes the C-terminal glycine of LC3 to bind to PE [31]. PE is conjugated to the C-terminal glycine of LC3-I, and this conjugation needs to be catalyzed by the E1-like enzymes ATG7 and E2-like enzymes ATG3 [32]. Therefore, after processing and modification, LC3-I binds to PE on the membrane surface of the autophagosome and becomes LC3-II in the form of membrane binding (Figure 4(b)). LC3-II is located in the proautophagosome and autophagosome and is an important autophagosome marker molecule that increases with autophagosome membrane augmentation [33]. The ratio of LC3-II/LC3-I or the concentration of LC3-II is positively correlated with the number of autophagosomes, reflecting the degree of autophagy activity of cells to a certain extent.

#### 2.3.4. Autophagy Adaptors Transport Autophagic Goods

LC3 can not only act as a sign of the autophagosome membrane, but it can also function as a docking site for a series of goods receptors to bring autophagic goods to autophagic vesicles. Goods receptors such as P62 (also known as sequestosome-1, SQSTM1) bind to LC3 and ubiquitinated substrates, which are subsequently integrated into the autophagosome and degraded in the autophagolysosome [34]. In addition, specific goods receptors preferentially bind to special goods, so goods receptors may provide selectivity for autophagy progress [35].

#### 2.3.5. Maturation of the Autophagosome

After autophagic vesicles form, an additional membrane will be delivered to form the vesicle and close it. The membrane derived from different organelles is recruited to form the autophagic vesicles by ATG9 [36, 37]. It has been proposed that the mammalian homolog of ATG9 shifts localization in membrane recruitment [38]. After the isolation membrane completely closes, the vesicle is named an autophagosome.

#### 2.3.6. Fusion of the Autophagosome-Lysosome and Lysosomal Degradation

With the formation of the autophagosome, it will fuse with the lysosome and become an autolysosome. Several proteins, such as VTI1B, syntaxin 17, WAMP8, RAB, and LAMP2, play key roles in the fusion process [39–42]. Finally, the goods are degraded by lysosomal proteases. And degradation products, including amino acids, fatty acids, and nucleotides, are recycled for further use in various kinds of metabolic processes [25].

### 2.4. Classification of Autophagy

**Figure 5:**
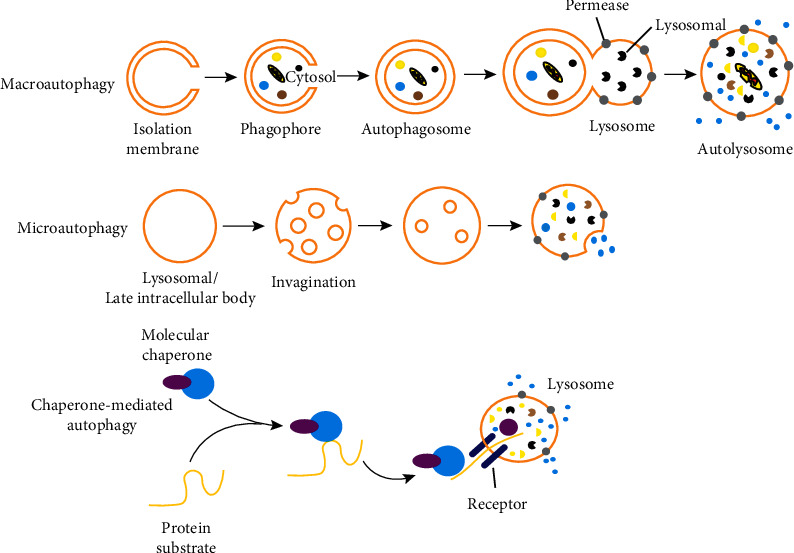
Three main types of autophagy. Macroautophagy depends on the formation of cytosolic phagophores to encapsulate and transport contents to the lysosome. Microautophagy absorbs the contents directly through the invagination of the lysosomal membrane. Chaperone-mediated autophagy transports the proteins directly across the membrane of the lysosome for digestion.

In addition to special lysosomal, ribosomal, and other selective autophagy, according to the types of substrates, modes of transport, and regulatory mechanisms, autophagy can be broadly divided into three categories, including microautophagy, macroautophagy, and chaperon-mediated autophagy (CMA) [43–45]. Macroautophagy, commonly referred to as autophagy, is one of the most widely studied types of autophagy and is an ancient and conserved self-degradation process. The main process of its action is that the cytoplasm is surrounded by nonribosomal regions of the endoplasmic reticulum, Golgi apparatus, and other peeling bilayers to remove organelles, pathogens, and protein aggregates and thus play a homeostasis role in normal cells [25]. Microautophagy is a nonselective lysosomal degradation process that involves autophagy tubes directly engulfing cytoplasmic contents on the boundary membrane, mediating invagination and vesicle rupture into the lumen. The main functions of microautophagy are to maintain organelle size, membrane homeostasis, and cell survival under nitrogen deficiency conditions. In addition, microautophagy is coordinated and supplemented with macroautophagy and CMA [46]. Autophagy mediated by CMA is the binding of intracytoplasmic proteins to molecular chaperones and then transporting them to the lysosomal cavity for digestion by lysosomal enzymes. The substrate of CMA is a kind of soluble protein molecule, so the CMA degradation pathway is selective in scavenging proteins, while the former two have no obvious selectivity [47] (Figure 5).

### 2.5. The Relationship between Autophagy and Tumors

**Figure 6:**
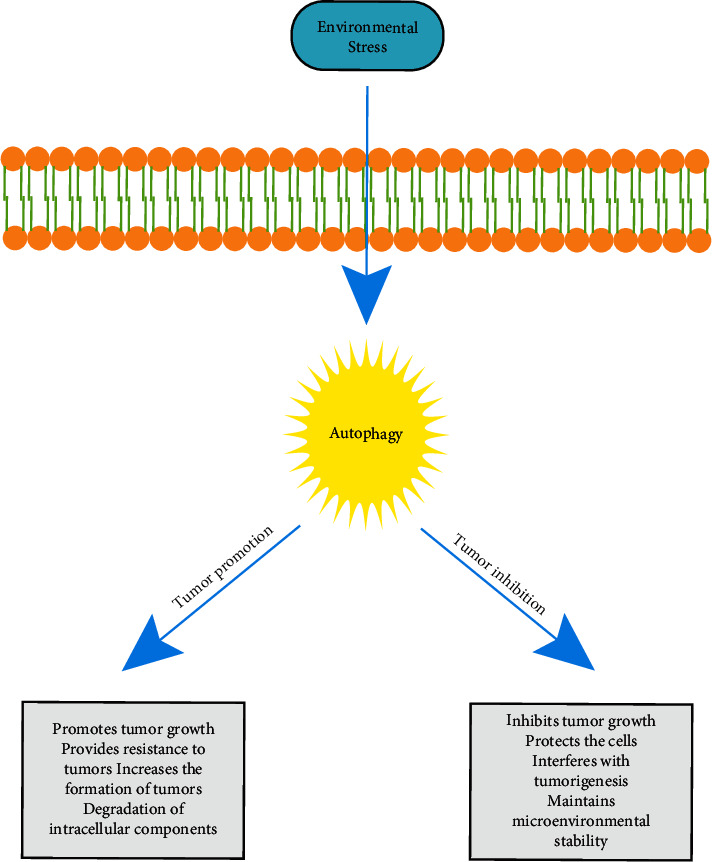
A schematic diagram of the autophagy roles of tumor promotion and inhibition in cancer cells.

A direct correlation between autophagy and tumors was first discovered in 1999 [48]. The potential role of autophagy in cancer is quite complex and is related to tumor induction and inhibition [12]. Tumor cells are more dependent on autophagy for survival than normal cells, partly because their rapid growth rates change the metabolic and nutrient-deficient growth and living environments. Some chemotherapeutic agents can regulate the process of autophagy, so autophagy-regulated chemotherapy can participate in cancer survival or death [49, 50]. Abnormal and decreased autophagy restrain the degradation of organelles or proteins in oxidative-stressed cells, resulting in the development of cancer. Besides, autophagy regulation can contribute to the expression of tumor suppressor-associated proteins or oncogenes.Tumor-inhibiting factors are negatively regulated by AMP-activated protein kinase (AMPK) and mammalian target of rapamycin (mTOR), leading to the formation of autophagy and the inhibition of cancer appearance [51]. Based on the dual role of autophagy in tumor genesis and development, inhibition of tumor growth by regulating autophagy activity has gradually become a new research field and direction of autophagy therapy for tumors (Figure 6). To fully understand the process and mechanism of autophagy is of great significance for the clinical treatment of cancer.

## 3. Mechanisms of MDR in Tumors

### 3.1. Mechanism Categories of MDR

Drug resistance of tumor cells is still one of the biggest obstacles to tumor chemotherapy. It is estimated that about 90 percent of chemotherapy failures are associated with drug-resistant migration and invasion of tumor cells [52]. The mechanisms of multiple drug resistance in tumors can be classified into the following categories: membrane transporters with ATP-binding cassette (ABC) transporters as the main transporters increase drug efflux [53]; DNA repair mechanisms are increased and drug-induced apoptosis is blocked [54]; internal circulation of transports, such as solute carriers, reduces drug absorption [55]; adaptability is enhanced through epigenetic regulation and miRNA regulation [56, 57]; mutations in the P53 pathway or changes in the expression levels of B-cell lymphoma (BCL) family proteins block the transmission of information among apoptosis signaling pathways in intracellular [58, 59]; elimination of glutathione S-transferase and cytochrome P450 enzymes promotes drug metabolism [60, 61]; drug target mutations or feedback effects of other targets and signaling pathways block drug-mediated tumor toxicity [62]; chemotherapeutic resistance is due to changes in the tumor microenvironment, such as tumor stem cell regulation and hypoxia response [63, 64]; both antiapoptosis of tumor cells and epithelial-mesenchymal transition are participated in tumor drug resistance [65–67]. The mechanisms of cellular drug resistance can be further subdivided into transporter-based classical and nonclassical MDR phenotypes.

### 3.2. ABC Transporters

The ABC superfamily contains 49 different types of transporters, which can be divided into 7 subfamilies from ABC-A to ABC-G according to sequence similarity and structural composition [68]. Among them, human ABCB1 is the earliest discovered ABC transporter. Many studies have confirmed that the overexpression of ABCB1/MDR1 is the main factor limiting the efficacy of chemotherapy drugs in vitro [69, 70]. The P-glycoprotein encoded by the MDR1 gene is the most widely studied ABC transporter. P-glycoprotein can use the energy released by ATP decomposition to transport various structurally and functionally unrelated drugs out of cells [71]. In addition to its expression in normal tissues, overexpression of P-glycoprotein can lead to the development of MDR in tumor cells [70]. Therefore, overcoming multiple resistances based on P-glycoprotein has been extensively studied for more than 30 years. ABC transporters are considered to be the main cause of MDR development. The goal of the continued development of antitumor therapy is to block or inactivate ABC transporters to increase intracellular anticancer drug concentration [72].

## 4. The Role of Autophagy in MDR Tumors

Many literature reports have suggested that autophagy is involved in the process of drug resistance. The research of Bhardwaj et al. [73] found that the expression of ATG5, LC3, and Beclin-1 was significantly increased in tumor tissues and was positively correlated with the expression level of the MDR1 gene. This suggests that autophagy is involved in the occurrence and development of MDR. However, some research evidence has emerged in recent years to show that the role of autophagy in tumorigenesis and development is complex and controversial. Autophagy plays an anticancer role in normal cells by removing damaged organelles, protein components, and circulating products. Paradoxically, excessive autophagy can lead cancer cells to develop “II type programmed cell death” or “autophagic cell death.” Autophagy plays a dual role in the occurrence and development of tumors and the resistance of tumor cells to chemotherapy [74]. Autophagy can be activated during antitumor therapy as a protective mechanism for MDR. Therefore, inhibition of autophagy can enhance the sensitivity of tumor cells to chemotherapy and thus enhance the cytotoxicity of chemotherapy drugs. However, autophagy may also induce autophagic cell death. Therefore, autophagy can improve the therapeutic effect of tumor MDR as long as it is properly applied in tumor treatment. The role of autophagy in MDR needs to be clarified.

### 4.1. Autophagy Protects MDR Tumors for Survival

#### 4.1.1. Autophagy Mediates MDR

The ABC transporter is closely related to the occurrence of MDR. Therefore, research advocates the development of drugs that regulate ABC transporters as chemotherapy agents to overcome MDR. However, ABC transporter modulators have not yet achieved ideal clinical therapeutic effects. In addition, there are many complex phenotypes in MDR. Recent studies have attempted to link autophagy with MDR based on the provided clinical data. In tumor specimens of colorectal cancer patients who survived for 5 years, the expression level of ABCB1 was positively correlated with the expression levels of Beclin1, LC3, Rictor, and negatively correlated with the expression level of Raptor [75]. It is suggested that autophagy is closely related to the occurrence and development of MDR.

#### 4.1.2. Autophagy Promotes the Generation and Development of MDR

Autophagy has been confirmed to promote tumor survival in lung cancer, esophageal cancer, liver cancer, ovarian cancer, kidney cancer, prostate cancer, stomach cancer, pancreatic cancer, breast cancer, colorectal cancer, bladder cancer, and glioma [76–87]. Research has shown that MDR is produced and formed after autophagy. The increased autophagy level in patients with poor prognosis suggests that the presence of autophagy may promote the development of MDR. MDR can be caused by a variety of factors, including the involvement of signaling pathways, various gene targets, and related proteins.

- Beclin1 enhances the production of MDR
- The scientific research of Lu et al. [88] showed that multiple myeloma (MM) patients with profilin1 (PFN1) expression had a poor prognosis. PFN1 can bind to the Beclin1 complex and promote the initiation of autophagy. The study found that overexpression of PFN1 not only promoted proliferation and bortezomib (BTZ) resistance but also facilitated the process of autophagy and induced BTZ resistance in MM. While inhibition of autophagy by blocking the formation of the Beclin1 complex can reverse BTZ resistance, the results suggest that PFN1 may contribute to BTZ resistance by participating in the Beclin1 complex to promote autophagy. Besides, MDR may be mediated by High-Mobility Group Box 1 (HMGB1). HMGB1 promotes autophagy in response to antitumor drugs. During its transfer from the nucleus to the cytoplasm, by activating the Mitogen-Activated Protein Kinase (MAPK)/Extracellular Signal-Regulated Kinase (ERK) signaling pathway to promote the formation of the Beclin1-class III phosphatidylinositol 3-kinase (Beclin1-PI3KC3) complex to induce autophagy [89]. In addition, HMGB1-induced autophagy participates in the chemoresistance of various kinds of tumors [90].
- Both ROS and autophagy contribute to the formation of MDR
- In most cases, tumor cells maintain homeostasis and support tumor growth by controlling stromal cell function and activating autophagy [91]. During tumorigenesis, cancer cells induce excessive production of reactive oxygen species (ROS), which activates stromal cells through oxidative stress response mechanisms and autophagy. Both autophagy activation and antioxidant defense mechanisms in the stroma can protect cancer cells from cell damage and death [91]. In addition, the autophagy-mediated matrix is rich in high-energy metabolite cycling, such as L-lactate and ketone bodies, which can support mitochondrial biosynthesis and tumor anabolism [91]. It has been reported that lactate and ketone bodies have the function of chemical inducers for tumor cells and can stimulate the growth and migration of tumor cells. Besides, compared with non-MDR tumor cells and normal cells, the ROS levels and the activity of scavenging/antioxidant enzymes in MDR cancer cells are increased [54, 92]. These results indicate that ROS is closely related to the MDR of tumor cells.

Autophagy-related MDR has become a challenge in cancer treatment. For example, autophagy promotes the resistance of human lung cancer cells to cisplatin, gefitinib, and erlotinib chemotherapy and facilitates the resistance of oral squamous cell carcinoma to cisplatin [93–96]. Others include temozolomide in glioblastoma; tamoxifen or trastuzumab in breast cancer; 5-fluorouracil (5-FU) in colorectal and esophageal cancer; and leukemia resistance to imatinib treatment [97–102].

#### 4.1.3. Inhibition of Autophagy Can Boost the Treatment of MDR Tumors

As a mechanism, autophagy facilitates the formation of resistance to chemotherapy, which weakens the curative effect of anticancer drugs. Thus, inhibition of autophagy may provide a potential tool to enhance the treatment efficiency of tumor cells. Current studies have found many kinds of autophagy suppression methods. Here, we list some of the common autophagy inhibition strategies.

- Application of autophagy inhibitors
- In much of the research, the use of autophagy inhibitors can increase the sensitivity of chemotherapeutic agents to tumor cells. Shi and Pan et al. showed that after the inhibition of autophagy by 3-methyladenine (3-MA), a PI3K inhibitor can enhance the cytotoxicity of cisplatin and 5-FU chemotherapeutic agents in nonsmall cell lung cancer [103, 104]. Another inhibitor, chloroquine (CQ), which blocks the fusion of autophagosomes with lysosomes, also has a similar autophagy inhibition effect. Research has shown that, under in vitro and in vivo conditions, the additional use of CQ enhances the inhibitory effect of 5-FU on the growth of tumor cells [100]. In the research of ovarian cancer SKVCR cells, the application of 3-MA and CQ increased the sensitivity of ovarian cancer MDR cells to chemotherapy by inhibiting autophagy, leading to an increase in cell apoptosis [105]. In human osteosarcoma and melanoma cells, these two inhibitors also increased the sensitivity to apoptosis induced by tumor necrosis factor-related apoptosis-inducing ligand (TRAIL) [106]. This inhibition of autophagy has also been shown to be beneficial with the use of other antitumor drugs, such as cisplatin, doxorubicin, and paclitaxel [68].
- Inhibition of ATGs
- In addition to autophagy inhibitors, inhibition of autophagy by gene silencing of ATG5, Beclin1, and other ATGs can enhance the sensitivity of MDR cells to chemotherapy drugs. For example, in osteosarcoma, miRNA-22 can inhibit the expression of ATG5, Beclin1, and LC3, which can enhance the sensitivity of tumor cells to chemotherapy [107]. In gastric cancer, miR-874 can inhibit autophagy to regulate the MDR of tumor cells by targeting ATG16L1 and enhance the sensitivity of tumor cells to chemotherapy drugs [108]. Autophagy inhibition by targeting ATG12 via small interfering RNAs (siRNAs) can also enhance the sensitivity of gastric cancer cells to chemotherapy drugs. All the above studies indicate that inhibition of autophagy can improve the utilization efficiency of chemotherapy drugs and reverse tumor MDR to a certain extent.
- Regulation of signaling pathways
- The efficiency of chemotherapeutic agents can be enhanced by regulating the signaling pathway and the effect of autophagy in MDR tumor cells. Huang et al. revealed the interaction between tyrosine kinase signals coordinated by the HGF-Met axis and autophagy, and the chemotherapy resistance of liver cancer can be reversed by regulating the HGF-Met axis and inhibiting autophagy [109]. In their study, Xin et al. [110] found that the inhibition of autophagy in drug-resistant gastric cancer cells by regulating the HULC/FOXM1 signaling pathway could reduce the drug resistance of the cells. In the research of ovarian cancer, targeting microRNA1301 can inhibit the proliferation of cisplatin-resistant ovarian cancer cells by inhibiting the NF-*κ*B signaling pathway and regulating autophagy, thus playing a defensive role in the occurrence and development of drug-resistant ovarian cancer cells [111]. All these studies indicate that autophagy and signaling pathways are one of the key factors affecting the chemotherapy of MDR tumor cells while regulating the signaling pathways and inhibiting autophagy are potential strategies to reverse cell MDR.

### 4.2. Autophagy Promotes the Death of MDR Tumors

#### 4.2.1. Autophagy Enhances the Sensitivity of MDR Cells to Chemotherapy

A large number of studies have shown that autophagy is one of the main causes of drug resistance in MDR tumor cells, so it can interfere with the toxic effects of chemotherapy drugs on tumors. It has also been proved that, on the basis of antitumor chemotherapy drugs, increasing autophagy can enhance drug efficacy and thus reverse MDR. For example, nanocrystal of underivatized fullerene C60 (Nano-C60) has cytotoxicity against human hepatoma cells [112]. Harhaji et al. [113] showed that the presence of intracytoplasmic vesicle acidification induced by Nano-60 in glioma cells suggested that autophagy contributed to the cell growth inhibition of Nano-C60. The induction of cell autophagy by Nano-C60 requires photoactivation to enhance the production of ROS. In Adriamycin-resistant MCF-7 breast cancer cell lines, autophagy production induced by Nano-C60 strengthens the chemotherapeutic sensitivity of Adriamycin-resistant tumor cells. Secondly, the chemosensitization effect of Nano-C60 is also dependent on autophagy, because the application of ROS scavenger N-acetyl-L-cysteine not only restrains the autophagy reaction induced by Nano-C60 but also significantly inhibits the chemosensitization effect of Nano-C60 [114]. Similarly, Nano-C60 has a variety of biological activities, including induction of autophagy and calcium/calmodulin-dependent protein kinase II*α* (CaMKII*α*). CaMKII*α* is a complex functional protein kinase that can promote the development process of tumor cells. Inhibition of its activity by the chemical inhibitor KN-93 or the knockout of CaMKII*α* can rapidly facilitate the antitumor activity of Nano-C60. Meanwhile, Nano-C60 can lead to alkalization and enlargement of lysosomes, impair their degradation function, and generate the accumulation of autophagosomes. Excessive accumulation of autophagosomes and inhibition of autophagy degradation play an important role in promoting osteosarcoma cell death. Therefore, inhibition of autophagy degradation can alter and promote the antitumor activity of Nano-C60 [115].

#### 4.2.2. Autophagy Enhances Cell Death in Apoptosis-Deficient MDR Tumors

In apoptosis-deficient MDR tumors, autophagy can generate adaptive responses in vivo that increase the resistance of MDR tumors to chemotherapeutic agents. However, under specific conditions, autophagy can eliminate other influencing factors in the cell apoptosis signaling pathways, making MDR tumors sensitive to apoptosis signals. In ABCB1-overexpressed and etoposide-resistant A549 lung cancer cells, interferon A can induce the formation of cell autophagy through the mTOR/Beclin1/ATG5 pathway. While autophagy inhibition by siRNA silencing Beclin1 can limit the occurrence of interferon A-induced apoptosis, further stimulation of autophagy under the effect of rapamycin can accelerate interferon A-induced apoptosis [116]. In TRAIL drug-resistant prostate cancer, cantharidin-induced production of autophagy can increase TRAIL-mediated apoptosis. Studies have shown that cantharidin could downregulate FADD-like IL-1*β*-converting enzyme (FLICE) inhibitor protein (C-FLIP), upregulate death receptor 5 (DR5), and enhance the level of autophagy, which ultimately strengthens cell apoptosis triggered by TRAIL [117].

#### 4.2.3. Multiple Factors Promote Autophagy-Mediated MDR Tumor Death

- Drug-mediated autophagy is involved in MDR tumor death
- The basic level of autophagy is the mechanism of tumor inhibition by reducing damaged cell parts and proteins and maintaining cell homeostasis [118]. In MDR tumor cells, autophagy can play a prodeath role and trigger autophagic cell death. Some studies are trying to find new antitumor drugs that attempt to kill MDR cells by inducing overautophagy. Kaewpiboon et al. [116] showed that the compound can overcome drug resistance by inducing autophagy, which in turn heightens the process of apoptosis. There is also some research thinking that autophagy helps to remove abnormal and damaged structures or harmful substances from normal cells, while failure to remove these substances can lead to the accumulation of mutations and other cancer-causing substances. Cryptotanshinone and dihydrotanshinone can inhibit the growth of antiapoptotic CRC cells by inducing autophagic cell death and P53-independent cytotoxicity [119]. In a leukemia cell line, drug-resistant cell K562, drug resistance can be antagonized by edifuxin lipid nanoparticles and induced caspase-independent autophagic cell death [120]. In cisplatin-resistant nonsmall cell lung cancer (NSCLC), baicalein can increase cisplatin-induced autophagy to overcome cell resistance and promote drug-resistant cell death [121]. An indole alkaloid drug, Voacamine, can also induce the occurrence of cell autophagy, which mediates the death of MDR tumor cells [122]. In addition, a newly developed drug, Suberoylanilide hydroxamic acid (SAHA), induces autophagic cell death in tamoxifen-resistant breast cancer cells MCF-7 and can significantly inhibit the proliferation of tumor cells [123].
- The complex role of drug-mediated autophagy on tumor cells
- In addition to drug-mediated autophagy-promoting drug-resistant cell death, some ATGs may also be involved in promoting MDR cell death. In antietoposide A549 cells, interferon A not only downregulated the expression of P-glycoprotein but also led to an increase in ATG5, LC3, and Beclin1. These changes are related to the increase in cell apoptosis. Besides, the inhibition of autophagy by taking advantage of siRNA can reduce cell apoptosis, while the level of cell apoptosis can be significantly increased with the addition of an autophagy inducer [116]. However, the failure to increase the level of cell apoptosis while inducing an increase in cell autophagy may also be related to the inhibition of tumor cell proliferation by autophagy. This view was confirmed in the study on the effect of metformin on myeloma cells, namely the expression of autophagy markers was enhanced while the expression of apoptosis-related factors was not found to be boosted when metformin acted on cells [124]. So far, the effects of drugs on tumor cells can be quite different under different conditions, which is still a confusion in many studies. For example, autophagy induced by cisplatin can promote the death of drug-resistant cells in NSCLC, while in oral squamous cell carcinoma, cisplatin-mediated autophagy has the effect of inducing drug resistance and thus protecting tumor cells [96, 121]. And this phenomenon can be changed by other conditions. Erkisa et al. [125] discovered that the combination of the autophagy inhibitor CQ and the barbiturate palladium (II) complex can enhance the sensitivity of tumor cells to chemotherapeutic drugs and strengthen the apoptosis rate of cells. Furthermore, it should be noted that it is not just chemotherapy drugs that are autophagy inducers; other factors also need to be seriously considered in the anticancer treatment process. According to Izdebska et al. [126], the application of lidocaine to rat glioma C6 cell lines can result in the production of protective autophagy.
- Signaling pathways participate in promoting the death of MDR tumors
- In MDR tumor cells, some signaling pathways may also be involved in autophagic cell death. The combination of sertraline and erlotinib can induce autophagy by regulating the AMPK/mTOR pathway, and the combination of the two drugs can significantly reduce the formation of tumors [127]. Phosphatidylinositol 3-kinase (PI3K) and the mTOR inhibitor NVP-BEZ235 inhibit the proliferation of cisplatin-resistant urothelium carcinoma cells by activating autophagy independent of apoptosis cell death [128]. In lung cancer cells, curcumin-induced autophagy is associated with the activation of the AMPK signaling pathway [129]. In addition, in human melanoma cells, Polygonatum cyrtonema lectin (PCL) induces autophagy through the mitochondria-associated ROS-p38-p53 pathway [130]. Other cellular signaling pathways may also be involved in the autophagic death of MDR cells. For example, the underlying mechanism of resveratrol-mediated activation of autophagic cell death may be highly dependent on the environment and cell type since it can influence the effect and function of many signaling pathways, including PI3K-AKT, DAPK1, Beclin1, STIM1-mTOR, WNT/*β*-Catenin, and so on [131–136]. On the contrary, in some research, the pathways have a negative regulatory effect on autophagy-promoting MDR cell death. Chen et al. showed that triptolide could increase the chemotherapy sensitivity of A549 cisplatin-resistant cells by inhibiting the PI3K/AKT/mTOR pathway to induce autophagy in lung cancer cells [137].

Although the exact mechanism of autophagy-mediated MDR cell sensitivity to chemotherapy drugs still needs to be further explored, the findings of the above research highlight the new function of autophagy in the reversal of MDR tumor cell therapy and provide a new plan and strategy for MDR therapy.

## 5. Conclusion and Prospects

Among the multiple treatment methods for tumors, the changes in radiotherapy, chemotherapy, and surgery have made significant progress in the past decades, but MDR is still an urgent problem to be solved in tumor chemotherapy. MDR of tumor cells can significantly inhibit the utilization efficiency of anticancer drugs and plays a key role in the successful treatment of tumors. The generation of MDR can be caused by a variety of factors, which can play an irreplaceable role in the reversal of tumor MDR therapy. The prosurvival and prodeath effects of autophagy may depend on the characteristics of tumor cells and the treatment methods. Autophagy can protect tumor cells to promote survival and can mediate the development of drug resistance in tumor cells during chemotherapy. While the inhibition of autophagy can not only enhance the sensitivity of MDR tumor cells to chemotherapy drugs, in some tumors, autophagy inhibitors can collaborate with chemotherapy drugs to jointly promote the apoptosis of tumor cells. This feature remains mysterious in much of the research on tumors and deserves further exploration. Research has shown that autophagy inhibitors CQ and 3-MA can enhance the cytotoxicity of chemotherapeutic agents when used in combination with some antitumor agents and enhance the sensitivity of MDR tumor cells to drugs. On the other hand, various factors in the cellular environment may affect the production of autophagy. Autophagy inducers mediate the production of autophagy, which can lead to autophagic cell death and eventually kill MDR cells, thus providing a new strategy for anti-MDR cell therapy. Besides, new studies have shown that autophagy can promote MDR cell death, make antiapoptotic MDR cells sensitive to anticancer drugs and reverse tumor cell MDR. This provides an ideal application prospect for autophagy in the treatment of MDR tumors and can overcome the MDR problems of tumor cells with the help of this function of autophagy. Although the molecular mechanism of the interaction between autophagy and tumor MDR has not yet been elucidated, these studies provide clues and solutions for us to further explore the relationship between them and leave a broad space for us to explore.
